# Prognostic factors in 448 newly diagnosed multiple myeloma receiving bortezomib-based induction: impact of ASCT, transplant refusal and high-risk MM

**DOI:** 10.1038/s41409-024-02227-0

**Published:** 2024-02-21

**Authors:** Hoi Ki Karen Tang, Chi Yeung Fung, Yu Yan Hwang, Harold Lee, Grace Lau, Sze Fai Yip, Bonnie Kho, Chi Kuen Lau, Kwan Hung Leung, Elaine Au, Eric Tse, Joycelyn Sim, Yok Lam Kwong, Chor Sang Chim

**Affiliations:** 1 Department of Medicine, Queen Mary Hospital, University of Hong Kong, Pok Fu Lam, Hong Kong; 2 Department of Medicine, Princess Margaret Hospital, Kwai Chung, Hong Kong; 3 Department of Medicine, Tuen Mun Hospital, Tuen Mun, Hong Kong; 4 Department of Medicine, Pamela Youde Nethersole Eastern Hospital, Chai Wan, Hong Kong; 5 Department of Medicine, Tseung Kwan O Hospital, Tseung Kwan O, Hong Kong; 6 Department of Medicine, United Christian Hospital, Kwun Tong, Hong Kong; 7 Department of Pathology, Queen Mary Hospital, University of Hong Kong, Pok Fu Lam, Hong Kong; 8 Department of Medicine, Hong Kong Sanatorium & Hospital, Happy Valley, Hong Kong

**Keywords:** Myeloma, Bone marrow transplantation

## Abstract

In Hong Kong, newly diagnosed multiple myeloma (NDMM) receives bortezomib-based triplet induction. Upfront autologous stem cell transplant (ASCT) is offered to transplant eligible (TE) patients (NDMM ≤ 65 years of age), unless medically unfit (TE-unfit) or refused (TE-refused). Data was retrieved for 448 patients to assess outcomes. For the entire cohort, multivariate analysis showed that male gender (*p* = 0.006), international staging system (ISS) 3 (*p* = 0.003), high lactate dehydrogenase (LDH) (*p* = 7.6 × 10^−7^) were adverse predictors for overall survival (OS), while complete response/ near complete response (CR/nCR) post-induction (*p* = 2.7 × 10^−5^) and ASCT (*p* = 4.8 × 10^−4^) were favorable factors for OS. In TE group, upfront ASCT was conducted in 252 (76.1%). Failure to undergo ASCT in TE patients rendered an inferior OS (TE-unfit *p* = 1.06 × 10^−8^, TE-refused *p* = 0.002) and event free survival (EFS) (TE-unfit *p* = 0.00013, TE-refused *p* = 0.002). Among TE patients with ASCT, multivariate analysis showed that age ≥ 60 (*p* = 8.9 × 10^−4^), ISS 3 (*p* = 0.019) and high LDH (*p* = 2.6 × 10^−4^) were adverse factors for OS. In those with high-risk features (HR cytogenetics, ISS 3, R-ISS 3), ASCT appeared to mitigate their adverse impact. Our data reaffirmed the importance of ASCT. The poor survival inherent with refusal of ASCT should be recognized by clinicians. Finally, improved outcome with ASCT in those with high-risk features warrant further studies.

## Background

High dose melphalan followed by autologous stem cell transplant (ASCT) has been the standard of care for eligible patients with newly diagnosed multiple myeloma (NDMM) for over two decades [1]. Even when novel agents are used in first line treatment, addition of upfront ASCT has consistently shown to improve progression free survival (PFS) [2–5] and overall survival (OS) [6, 7]. Nonetheless, MM treatment is still challenged by relapses, precluding long-term remissions and hence cure of disease. Factors such as lack of response, International Staging System (ISS) stage, and high-risk cytogenetics have been linked to poor outcome in patients undergoing ASCT [8–17]. Other additional factors such as age, comorbidities, and cognitive/physical conditions have also been described to affect survival in ASCT patients [18–21].

In Hong Kong, bortezomib based triplet induction therapy (VTD/VCD) is given to patients with NDMM and upfront ASCT is generally offered to all consenting myeloma patient aged ≤65 years. Patients >65 years are considered transplant-ineligible (TIE). Upfront ASCT is offered to all TE patients ≤65 years, unless patients are medically unfit (TE-unfit) or refused (TE-refused). Utilizing our database on patients receiving bortezomib-based induction therapy and information on ASCT status, risk factors for survival were analyzed for the entire cohort of NDMM patients comprising TE and TIE patients in addition to those who had undergone ASCT. Moreover, the impact of TE-unfit and TE-refused ASCT on clinical outcomes in a real-world context was studied. Furthermore, in patients with complete cytogenetic data, the impact of ASCT to overcome high risk features including high risk fluorescence in situ hybridization test (HR FISH), ISS 3 and Revised International Staging System (R-ISS) 3 were analyzed.

## Methods

### Patients and data collection

Symptomatic NDMM patients treated in seven hematology centers, including Queen Mary Hospital, Princess Margaret Hospital, Tuen Mun Hospital, Pamela Youde Nethersole Eastern Hospital, United Christian Hospital, Tseung Kwan O Hospital and Queen Elizabeth Hospital, from January 2006 to January 2020 in Hong Kong were included in this retrospective study. Demographics and disease characteristics at the time of diagnosis, as well as treatment and transplant information were retrieved from the electronic medical records with approval from the Institutional Review Board of the University of Hong Kong.

### Patient treatment

All patients received induction treatment containing bortezomib. The combination of choice with bortezomib was mainly determined by the drug availability at the time of diagnosis, patient tolerability and affordability.

Regarding patient selection in the TE and TIE groups, TE patients were recruited into the database consecutively over time as reimbursement for upfront bortezomib in TE patients is provided by our medical care. On the other hand, reimbursement for upfront bortezomib in TIE patients was not made available till 2019, and thus most TIE patients that could afford self-financed bortezomib upfront were included in this database. Patients deemed TE by their respective hematology centers received high dose melphalan 200 mg/m^2^ for their ASCT. None of the patients received reduced dose melphalan for conditioning.

### Definitions and clinical outcome variables

Clinical staging was based on the ISS and R-ISS. Complete remission (CR) was defined as complete resolution of disease with absent paraprotein, as evidenced by a negative serum protein electrophoresis (SPE) and immunofixation, and <5% plasma cells in the bone marrow. Near-complete remission (nCR) was defined as a negative SPE but positive immunofixation. An event included reappearance of the paraprotein on immunofixation in CR patients, recurrent paraproteinemia in the nCR patients, ≥25% paraprotein increase and/or appearance of new bone lesions. For patients with light chain myeloma, CR was defined as normalization of the level and ratio of serum free light chain, and negative serum and urine immunofixation. Herein, CR and nCR were grouped together as CR/nCR as immunofixation and bone marrow exam were not performed on all patients after reaching negative SPE.

### Fluorescence in situ hybridization (FISH)

Detection of cytogenetic aberrations was performed on myeloma cells in the bone marrow sample by FISH. Enrichment for myeloma cells was achieved by sorting with CD138 immunomagnetic beads (MiniMACS, Miltenyi Biotec, Auburn, CA). The FISH probes comprised of the IGH/FGFR3 dual color dual fusion translocation probe for detection of t(4;14) (p16;q32), the IGH/MAF dual color dual fusion translocation probe for detection of t(14;16)(q32;q23) and the TP53/CEP17 deletion color probe for the detection of p53 deletion. At least 200 nuclei were analyzed. The cut-off for positivity was above 10% for fusion or break apart probes and 20% for numerical abnormalities in accordance with 2012 European Myeloma Network interphase FISH consensus [22]. HR FISH was defined according to International Myeloma Working Group (IMWG)-defined HR FISH alterations [7], including t(4;14), t(14;16) or del(17p) [23]. As complete FISH data was only available in 277 patients, the prognostic impact of HR FISH and R-ISS would be analyzed in univariate but not multivariate analysis.

### Statistical analysis

Statistical analysis was conducted using IBM SPSS Statistics Version 26. Overall survival (OS) was measured from the date of treatment to the date of death or last follow-up. Event-free survival (EFS) was calculated from the date of treatment to the date of progression, relapse, death or last follow-up. The survival curves for OS and EFS were plotted using Kaplan-Meier method and compared by log-rank test. Multivariate Cox regression was performed to analyze the impact of risk factors on OS including diagnostic clinical parameters including gender, age, ISS stage, lactate dehydrogenase (LDH), immunoglobulin isotype, treatment response (post-induction CR/nCR) and ASCT for entire cohort, while ASCT was removed for transplanted cohort. Data on high-risk FISH was only available in 277 patients (61.8%), hence included in univariate but not multivariate analysis. A *p*-value of <0.05 was considered statistically significant and all p-values were two-sided.

## Results

The entire NDMM cohort comprised 448 patients, 331 TE and 117 TIE patients. In our entire cohort, the majority of patients received bortezomib/ thalidomide/ dexamethasone (VTd) treatment (*n* = 348, 77.7%). Others received bortezomib/ doxorubicin/ dexamethasone (PAd) (*n* = 30, 6.7%), bortezomib/ cyclophosphamide/ dexamethasone (VCd) (*n* = 26, 5.8%), bortezomib/ dexamethasone (Vd) (*n* = 23, 5.1%), bortezomib/ melphalan/ prednisolone (VMP) (*n* = 12, 2.7%) and bortezomib/ lenalidomide/ dexamethasone (VRd) (*n* = 9, 2%). There were no differences in the demographics and proportion of high-risk MM (HR MM) (ISS 3, high LDH, IgG isotype, HR FISH and R-ISS 3) between TE and TIE MM patients (Table 1).

**Table 1:** Comparison of risk factors between TE and TIE groups.

Risk Factors	TE	TIE	Chi-square *P*-value
Gender	Male: *N* = 183 (55.3%) Female: *N* = 148 (44.7%)	Male: *N* = 70 (59.8%) Female: *N* = 47 (40.2%)	0.448
ISS3	ISS3: *N* = 144 (44.7%) ISS1/2: *N* = 178 (55.3%)	ISS3: *N* = 60 (52.2%) ISS1/2: *N* = 55 (47.8%)	0.192
Hi-LDH	>ULN: *N* = 96 (32.2%) ≤ULN: *N* = 202 (67.8%)	>ULN: *N* = 41 (40.2%) ≤ULN: *N* = 61 (59.8%)	0.149
IgG Isotype	IgG: *N* = 166 (50.2%) Others: *N* = 165 (49.8%)	IgG: *N* = 50 (42.7%) Others: *N* = 67 (57.3%)	0.197
HR FISH	HR: *N* = 56 (27.3%) SR: *N* = 149 (72.7%)	HR: *N* = 18 (25.0%) SR: *N* = 54 (75.0%)	0.759
R-ISS3	R-ISS3: *N* = 75 (28.6%) R-ISS1/2: *N* = 187 (71.4%)	R-ISS3: *N* = 32 (33.7%) R-ISS1/2: *N* = 63 (66.3%)	0.363

*Hi-LDH* high lactate dehydrogenase, *HR FISH* high risk fluorescence in situ hybridization, *ISS* international staging system, *R-ISS* revised international staging system, *SR* standard risk, *TE* transplant eligible, *TIE* transplant ineligible, *ULN* upper limit of normal.

**Fig. 1:**
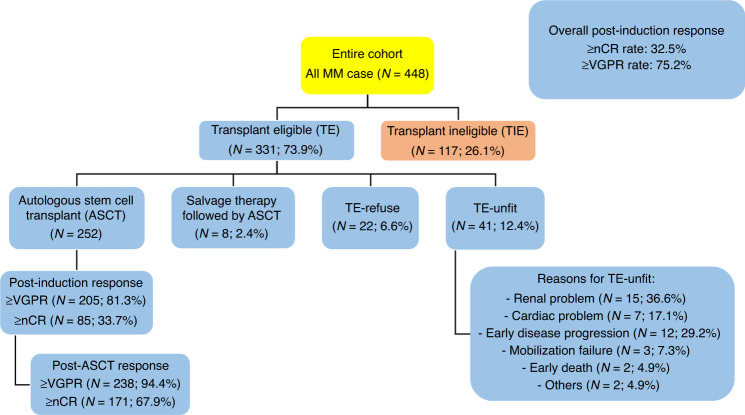
Flow chart of the study including patient’s subgroup and response rates. MM multiple myeloma, nCR near complete response, VGPR very good partial response.

The median age was 59.5 years old. One-hundred and forty-four (32.5%) patients achieved CR/nCR after induction and 189 (42.7%) patients achieved VGPR. Among TE patients, 260 (78.5%) underwent ASCT. Of those that received ASCT, 252 ASCT were performed after upfront bortezomib based induction and eight ASCT as consolidation after salvage therapy (Fig. 1). Among the 277 patients with complete FISH data, 74 (26.7%) patients had at least one HR FISH.

### Response after Induction and ASCT in transplanted MM

**Fig. 2:**
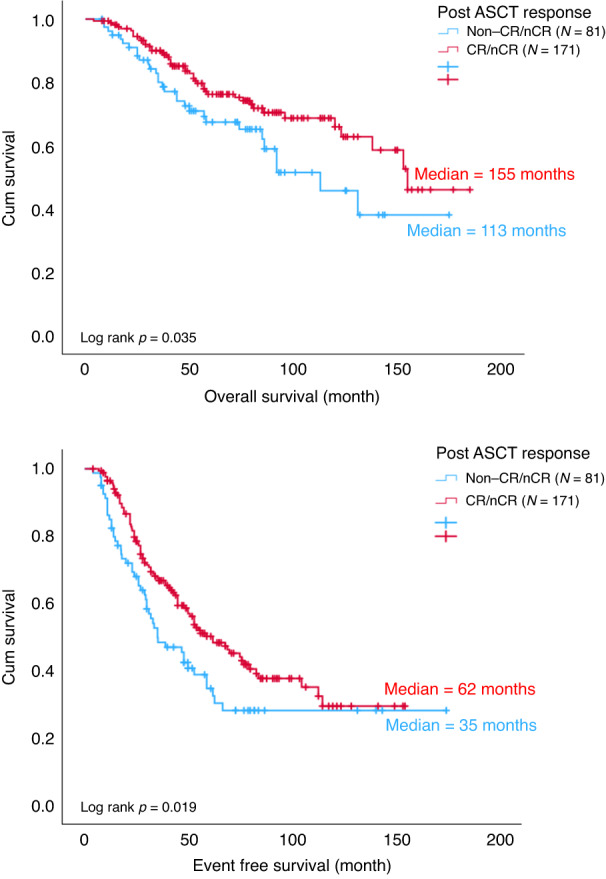
OS and EFS by post ASCT response (CR/nCR). ASCT autologous stem cell transplant, CR complete response, EFS event free survival, nCR near complete response, OS overall survival.

**Fig. 3:**
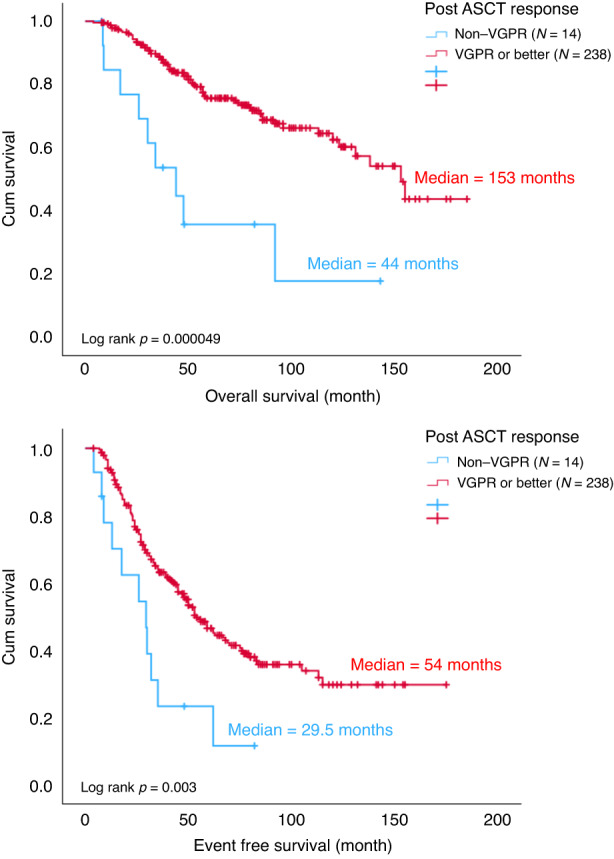
OS and EFS by post ASCT response (VGPR or better). ASCT autologous stem cell transplant, EFS event free survival, OS overall survival, VGPR very good partial response.

Among ASCT group, 85 (33.7%) patients attained CR/nCR after induction treatment and 171 (67.9%) patients achieved CR/nCR post ASCT. Two hundred and five (81.3%) patients attained VGPR or better after induction treatment and 238 (94.4%) patients achieved VGPR or better post ASCT (Fig. 1). After ASCT, the OS and EFS were superior in those that reached ≥CR/nCR (OS *p* = 0.035, EFS *p* = 0.019) or ≥VGPR (OS *p* = 0.000049, EFS *p* = 0.003) compared with those that failed to reach these end points after ASCT (Figs. 2 and 3).

### Predictors of EFS and OS in the entire cohort

The median OS was 113 months and median EFS 40 months for the entire cohort. Adverse risk factors for both EFS and OS include male gender (EFS *p* = 0.017, OS *p* = 0.003), ISS 3 (EFS *p* = 0.003, OS *p* = 2.2 × 10^−8^), R-ISS stage 3 (EFS *p* = 0.000001, OS *p* = 7.8 × 10^−13^), high LDH (EFS *p* = 0.000149, OS *p* = 2.8 × 10^−9^) (Table 2). Multivariate analysis (excluding HR FISH as data was unavailable in 171 patients within the entire cohort) showed that male gender (*p* = 0.006), ISS 3 (*p* = 0.003) and high LDH (*p* = 7.6 × 10^−7^) were negative predictors for OS. Achievement of post induction CR/nCR was a predictor of improved EFS and OS in univariate (EFS *p* = 7.3 × 10^−7^, OS *p* = 0.001) and multivariate analysis (OS *p* = 2.7 × 10^−5^). Patients who underwent ASCT had superior EFS (*p* = 0.000007) and OS (*p* = 7 × 10^−14^) in univariate and multivariate analysis (OS *p* = 4.8 × 10^−4^) (Tables 2 and 3) compared to those that did not receive ASCT (i.e. TIE, TE-refused and TE-unfit). Among the 277 patients with complete FISH data, the presence of HR FISH negatively impacted OS (*p* = 0.001) and EFS (*p* = 0.015) (Table 2).

**Table 2:** Prognostic factors for overall survival (OS) and event-free survival (EFS).

		*N* (%)	Median OS (months)	*P*-value	Median EFS (months)	*P*-value
Transplant eligibility	Ineligible	117 (26.1)	48	9.1 × 10^−8^	29	0.002
	Eligible	331 (73.9)	138		47	
Gender	Male	253 (56.5)	86	0.003	36	0.017
	Female	195 (43.5)	153		48.5	
ISS	3	204 (46.7)	55	2.2 × 10^−8^	30	0.003
	1/2	233 (53.3)	138		50	
R-ISS	3	107 (30.0)	42	7.8 × 10^−13^	22	0.000001
	1/2	250 (70.0)	131		50	
LDH	>ULN	137 (34.2)	48	2.8 × 10^−9^	26	0.000149
	≤ULN	263 (65.8)	155		50	
Isotype	IgG	216 (48.2)	120	0.023	48	0.083
	IgA	98 (21.9)	61		36	
	IgM	2 (0.4)	20		18	
	IgD	16 (3.6)	59		22	
	Light chain	107 (23.9)	90		40	
	Non-secretory	9 (2.0)	NR		69	
Induction Response	Non-CR	299 (67.5)	86	0.001	32	7.3 × 10^−7^
	CR/nCR	144 (32.5)	155		80	
HR FISH	High risk	74 (26.7)	61	0.001	25	0.015
	Standard risk	203 (73.3)	123		48	
ASCT	Yes	260 (58.4)	153	7 × 10^−14^	53	0.000007
	No	185 (41.6)	47		26	

*ASCT* autologous stem cell transplant, *CR* complete response, *HR FISH* high risk fluorescence in situ hybridization, *ISS* international staging system, *LDH* lactate dehydrogenase, *nCR* near complete response, *NR* not reached, *R-ISS* revised international staging system, *ULN* upper limit of normal.

**Table 3:** Multivariate analysis of prognostic factors for OS (384 patients with complete data in the entire cohort).

		Univariate analysis	Multivariate analysis		
		*P*-value	*P*-value	Hazard ratio	95% CI
Age		/	0.122	1.017	0.996–1.038
Gender	Male	0.003	0.006	1.643	1.152–2.341
	Female		Ref.	Ref.	
Isotype	IgA	0.023	0.015	1.712	1.109–2.641
	IgM		0.128	4.785	0.639–35.855
	IgD		0.112	1.836	0.867–3.889
	Light chain		0.828	0.953	0.620–1.466
	Non–secretory		0.961	0.965	0.225–4.130
	IgG		Ref.	Ref.	
ISS	3	2.2 × 10^−8^	0.003	1.71	1.195–2.448
	1/2		Ref.	Ref.	
LDH	>ULN	2.8 × 10^−9^	7.6 × 10^−7^	2.416	1.703–3.428
	≤ULN		Ref.	Ref.	
Induction Response	CR/nCR	0.001	2.7 × 10^−5^	0.422	0.282–0.632
	Non–CR		Ref.	Ref.	
ASCT	Yes	7 × 10^−14^	4.8 × 10^−4^	0.483	0.320–0.727
	No		Ref.	Ref.	

High risk FISH not included due to limited cases with FISH done at diagnosis

*ASCT* autologous stem cell transplant, *CI* confidence interval, *CR* complete response, *ISS* international staging system, *LDH* lactate dehydrogenase, *nCR* near complete response, *OS* overall survival, *Ref* reference, *ULN* upper limit of normal

### Predictors of EFS and OS in the ASCT cohort

A total of 260 patients received ASCT after bortezomib based induction with median OS of 153 months and median EFS 53 months. There were significant worsening of OS and EFS among those with ISS 3 (EFS *p* = 0.015, OS *p* = 0.000293), R-ISS stage 3 (EFS *p* = 0.000014, OS *p* = 9.5 × 10^−9^), age ≥60 (EFS *p* = 0.073, OS *p* = 0.002) and high LDH (EFS *p* = 0.027, OS *p* = 0.000025) (Table 4). Multivariate analysis (excluding HR FISH as there was incomplete data in 94 patients) showed age ≥60 (*p* = 8.9 × 10^−4^), ISS 3 (*p* = 0.019) and high LDH (*p* = 2.6 × 10^−4^) were adverse factors for OS (Table 5). Post induction CR/nCR correlated with superior EFS (EFS *p* = 0.006) but not OS (*p* = 0.154) in ASCT patients (Table 4). Among the 166 patients with complete FISH data post ASCT, the presence of HR FISH negatively impacted on OS (*p* = 0.023) but not EFS (*p* = 0.207) (Table 4).

**Table 4:** Prognostic factors for OS and EFS in TE myeloma who had undergone ASCT.

		*N* (%)	Median OS (months)	*P*-value	Median EFS (months)	*P*-value
Age	≥60 years old	83 (31.9)	92	0.002	42	0.073
	<60 years old	177 (68.1)	155		58	
Gender	Male	141 (54.2)	131	0.022	47	0.07
	Female	119 (45.8)	NR		62	
ISS	3	96 (37.9)	113	0.000293	35	0.015
	1/2	157 (62.1)	NR		59	
R-ISS	3	45 (21.7)	48	9.5 × 10^−9^	27	0.000014
	1/2	162 (78.2)	NR		55	
LDH	>ULN	64 (27.5)	80	0.000025	33	0.027
	≤ULN	169 (72.5)	NR		58	
Isotype	IgG	141 (54.2)	138	0.145	53	0.182
	IgA	49 (18.8)	NR		77	
	IgM	0 (0)	–		-	
	IgD	10 (3.8)	86		24	
	Light chain	53 (20.4)	NR		45	
	Non-secretory	7 (2.7)	NR		113	
Induction Response	Non-CR	175 (67.3)	138	0.154	45	0.006
	CR/nCR	85 (32.7)	155		83	
HR FISH	High risk	44 (26.5)	86	0.023	29.5	0.207
	Standard risk	122 (73.5)	131		48	

*ASCT* autologous stem cell transplant, *CR* complete response, *EFS* event free survival, *HR FISH* high risk fluorescence in situ hybridization, *ISS* international staging system, *LDH* lactate dehydrogenase, *nCR* near complete response, *NR* not reached, *OS* overall survival, *R-ISS* revised international staging system, *TE* transplant eligible, *ULN* upper limit of normal.

**Table 5:** Multivariate analysis of prognostic factors for OS in TE myeloma who had undergone ASCT (226 patients with complete data).

		Univariate analysis	Multivariate analysis		
		*P*-value	*P*-value	Hazard ratio	95% CI
Age	≥60 years old	0.002	8.9 × 10^−4^	2.339	1.417–3.859
	<60 years old		Ref.	Ref.	
Gender	Male	0.022	0.02	1.841	1.100–3.081
	Female		Ref.	Ref.	
Isotype	IgA	0.145	0.341	1.426	0.687–2.963
	IgM		/	/	/
	IgD		0.072	2.286	0.929–5.623
	Light chain		0.541	1.224	0.641–2.337
	Non-secretory		0.562	0.545	0.070–4.241
	IgG		Ref.	Ref.	
ISS	3	0.000293	0.019	1.81	1.103–2.972
	1/2		Ref.	Ref.	
LDH	>ULN	0.000025	2.6 × 10^−4^	2.495	1.528–4.075
	≤ULN		Ref.	Ref.	
Induction Response	CR/nCR	0.154	0.234	0.698	0.385–1.263
	Non-CR		Ref.	Ref.	

*ASCT* autologous stem cell transplant, *CR* complete remission, *CI* confidence interval, *ISS* international staging system, *LDH* lactate dehydrogenase, *nCR* near complete response, *OS* overall survival, *Ref* reference, *TE* transplant eligible, *ULN* upper limit of normal.

We further investigated the baseline characteristic and treatment responses of ASCT patients <60 and ≥60 years old, to explain for the survival difference within a narrow age spectrum. No differences were observed for gender, ISS, R-ISS, LDH, presence of IMWG-defined HR FISH, post induction CR/nCR, post induction VGPR, post ASCT CR/nCR and post ASCT VGPR between the two age groups (Table 6).

**Table 6:** Comparison of characteristics between transplanted patients <60 and ≥60 years old.

Characteristic		<60 years old		≥60 years old		Total		Chi-square *P*-value
Age	Total	177		83				/
	Median	54 years old		62 years old				
	Range	32–59 years old		60–68 years old				
Gender	Male	95	53.7%	46	55.4%	141	54.2%	0.849
	Female	82	46.3%	37	44.6%	119	45.8%	
ISS	3	65	38.2%	31	37.3%	96	37.9%	1.000
	1/2	105	61.8%	52	62.7%	157	62.1%	
R-ISS	3	28	20.9%	17	23.3%	45	21.7%	0.726
	1/2	106	79.1%	56	76.7%	162	78.3%	
LDH	>ULN	49	30.6%	15	20.5%	64	27.5%	0.117
	≤ULN	111	69.4%	58	79.5%	169	72.5%	
HR FISH	HR	24	22.4%	20	33.9%	44	26.5%	0.141
	SR	83	77.6%	39	66.1%	122	73.5%	
Post-induction CR/nCR	CR/nCR	62	35.0%	23	27.7%	85	32.7%	0.260
	Non-CR/nCR	115	65.0%	60	72.3%	175	67.3%	
Post-induction ≥VGPR	≥VGPR	143	80.8%	64	77.1%	207	79.6%	0.512
	<VGPR	34	19.2%	19	22.9%	53	20.4%	
Post-ASCT CR/nCR	CR/nCR	122	69.3%	52	62.7%	174	67.2%	0.322
	Non-CR/nCR	54	30.7%	31	37.3%	85	32.8%	
Post-ASCT ≥VGPR	≥VGPR	166	94.3%	78	94.0%	244	94.2%	1.000
	<VGPR	10	5.7%	5	6.0%	16	5.8%	

*ASCT* autologous stem cell transplant, *CR* complete response, *HR FISH* high risk fluorescence in situ hybridization, *ISS* international staging system, *LDH* lactate dehydrogenase, *nCR* near complete response, *R-ISS* revised international staging system, *SR* standard risk, *ULN* upper limit of normal, *VGPR* very good partial response.

### Survival analysis in the TE group

**Fig. 4:**
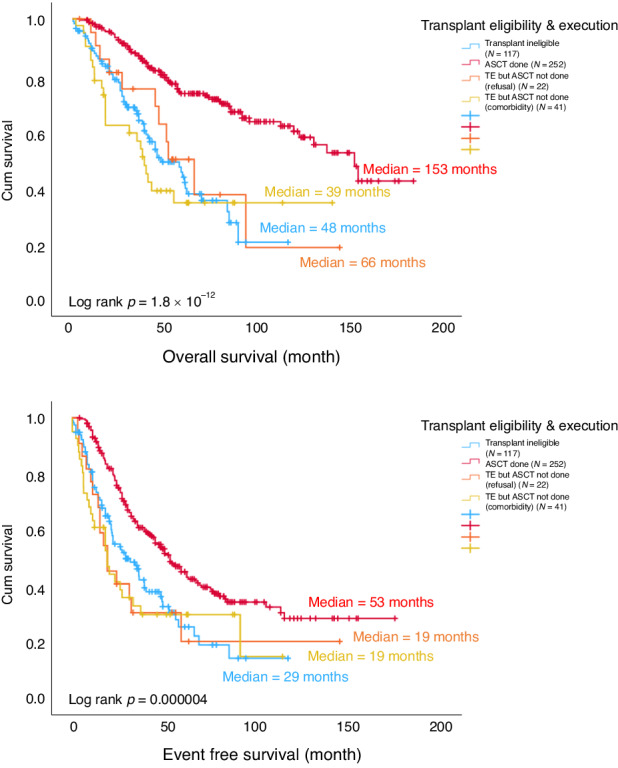
OS and EFS among patients in ASCT group (red), TE unfit (yellow), TE refused (orange) and TIE (blue). ASCT autologous stem cell transplant, EFS event free survival, OS overall survival, TE transplant eligible, TIE transplant ineligible.

Among the TE patients, ASCT was not performed in 63, due to being medically unfit (TE-unfit) (*N* = 41; 12.4%) or patient refusal (TE-refused) (*N* = 22; 6.6%). Major reasons patients were deemed unfit included renal impairment (*n* = 15, 36.6%), early disease progression (*n* = 12, 29.2%) and cardiac disease (*n* = 7, 17.1%) (Fig. 1). Compared with those transplanted MM, failure to undergo ASCT rendered a much inferior OS in TE-unfit (*p* = 1.06 × 10^−8^) and TE-refused (*p* = 0.002) and EFS (TE-unfit *p* = 0.00013, TE refused *p* = 0.002), with poor EFS and OS comparable to that of TIE patients (OS *p* = 0.576, EFS *p* = 0.614) (Fig. 4).

### Impact of ASCT in high-risk MM with IMWG-defined HR FISH, ISS3 or R-ISS

**Fig. 5:**
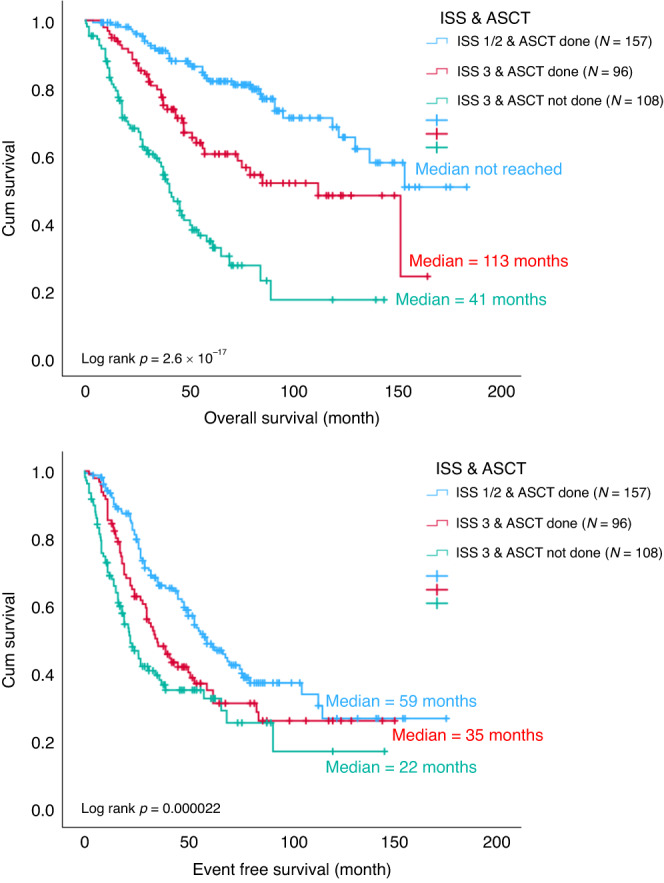
OS and EFS of ISS 1/2 patients with ASCT (blue), ISS 3 patients with ASCT (red) and ISS 3 patients without ASCT (green). ASCT autologous stem cell transplant, ISS international staging system, EFS event free survival, OS overall survival.

**Fig. 6:**
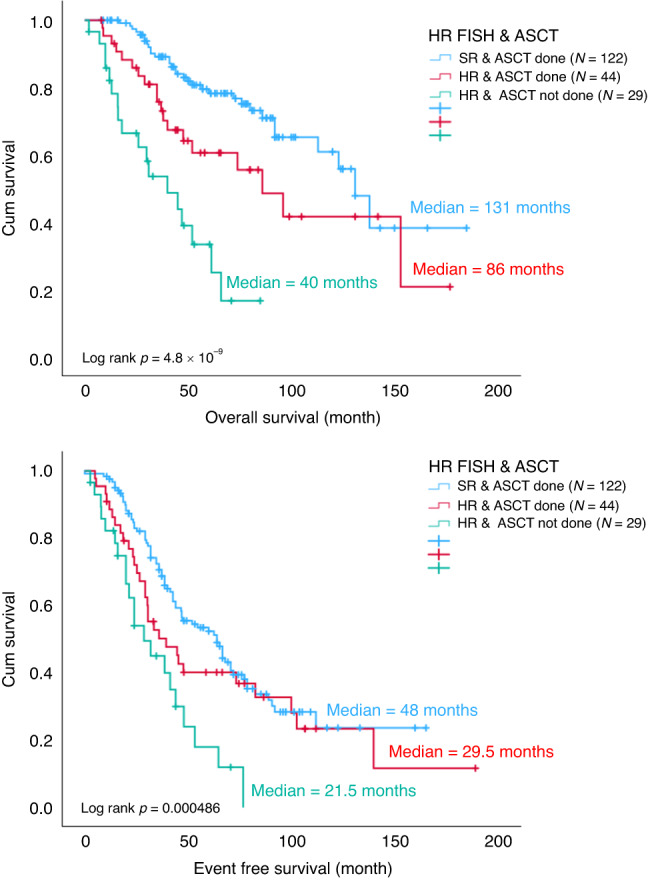
OS and EFS of SR FISH patients with ASCT (blue), HR FISH patients with ASCT (red) and HR FISH patients without ASCT (green). ASCT autologous stem cell transplant, EFS event free survival, HR FISH high risk fluorescence in situ hybridization, OS overall survival, SR standard risk.

**Fig. 7:**
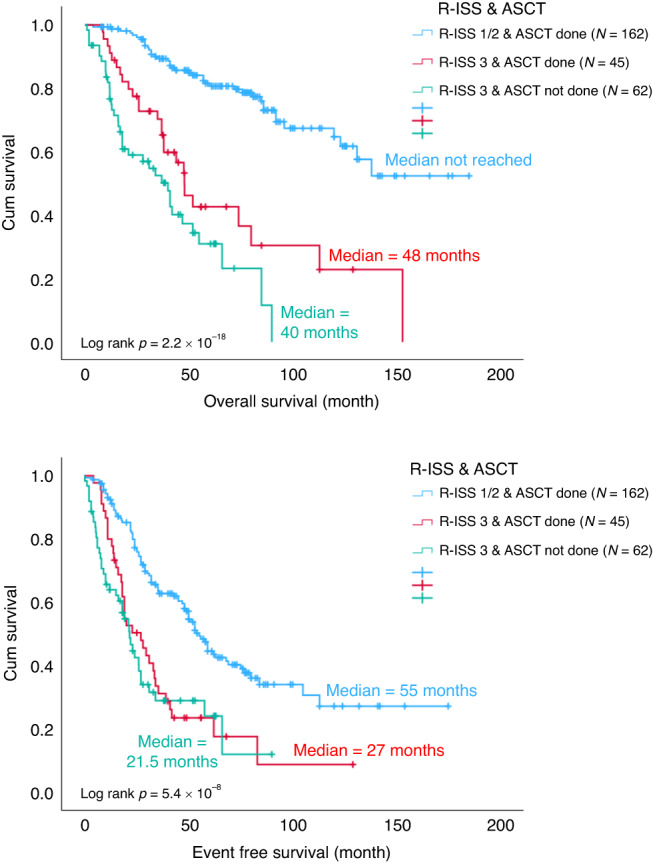
OS and EFS of R-ISS 1/2 patients with ASCT (blue), R-ISS 3 patients with ASCT (red) and R-ISS 3 without ASCT (green). ASCT autologous stem cell transplant, EFS event free survival, OS overall survival, R-ISS revised international staging system.

Among the entire cohort, 204 patients were ISS 3, of which 96 received ASCT. Compared to the ISS 1/2 patients with ASCT, the OS (*p* = 0.000293) and EFS (*p* = 0.015) were significantly inferior for those with ISS 3 and ASCT (Fig. 5). HR FISH abnormalities were found in 74 patients throughout the entire cohort, of which 44 received ASCT. Compared with standard risk FISH patients who underwent ASCT, the OS (*p* = 0.023) was still significantly worse in those with HR FISH despite receiving ASCT, though the EFS (*p* = 0.207) was comparable (Fig. 6). One hundred and seven patients from the entire cohort were R-ISS 3, of which 45 had ASCT done. Again, compared with R-ISS 1/2 patients who had ASCT, the OS (*p* = 9.5 × 10^−9^) and EFS (*p* = 0.000014) were significantly worse for those with R-ISS 3 even after ASCT (Fig. 7). The EFS and OS were the worst among those with either ISS stage 3, HR FISH and R-ISS stage 3 that did not receive ASCT (Figs. 5–7).

## Discussion

Our data reaffirms the real-world survival benefit of ASCT as well as the adverse impact of ISS 3, elevated LDH, HR FISH, transplant ineligibility and failure of achieving deep responses such as CR/nCR. These findings are consistent with previous publications on predictors of survival in NDMM patients [4, 5, 8–15, 24]. The EMN02/ HO95 trial using an induction regimen of VMP similar to ours showed significant improvement in PFS and OS among those transplanted [2]. Importantly, ASCT can deepen post induction response. There was a substantial increase in patients reaching CR/nCR and VGPR after ASCT that translated to significant improvement in EFS and OS among those reaching CR/nCR or VGPR post ASCT in our cohort. Therefore, a deep response is pivotal to superior survival. In this connection, minimal residual disease (MRD) may pose as a more important end-point of MM treatment [25, 26]. Indeed, MRD-negativity has been shown to render superior survivals [27–29]. Addition of a CD38 antibody has been shown to yield a higher rate of MRD negativity CR that translated into superior PFS [30–32]. Indeed, triple negative CR including immunofixation, MRD and acetate positron emission tomography negative CR maybe the next goal and definition of CR in MM treatment [33]. Moreover, to enhance the rate of deep response in non-transplant candidates, incorporation of CD38 antibody has been shown to render a higher rate of MRD negative CR that translated into superior PFS and OS than the control arm [34]. In a resource restricted setting, three weekly daratumumab is a cost-effective yet efficacious option compared to the weekly and biweekly loading regimen [35]. Despite age difference between TE and TIE patients, our data confirmed similar frequency of HR factors in both groups, showing that these factors in myeloma patients are independent of transplant eligibility. Irrefutably, comorbidities and frailty also play an important role in the response and overall prognosis of TIE patients. Myeloma specific comorbidity index and frailty score can be used more widely to predict mortality and treatment toxicities in older myeloma patients in clinical practice [19, 36].

Secondly, ASCT appears to mitigate but not abolish the adverse impact of HR FISH, with improvements with EFS but the OS remained inferior to SR MM undergoing ASCT. The mitigation of adverse prognosis in HR FISH patients is dependent not only on whether ASCT was performed but also the cumulative number of unfavorable risk factors present and the induction regimen given. Our induction regimen consisted mainly of VTd/ VCd before daratumumab was available, which in this day and age, is clearly not potent enough for high-risk patients. The FORTE trial comparing carfilzomib lenalidomide dexamethasone (KRd) plus ASCT versus carfilzomib cyclophosphamide dexamethasone (KCd) plus ASCT versus KRd alone showed a favorable impact of KRd plus ASCT in patients with one HR FISH compared with the other two arms. In double hit myeloma, the PFS and OS were significantly worse compared with SR and one HR FISH groups regardless of the treatment arms [4]. Potent induction regimen with Dara KRd plus ASCT in the single arm phase 2 MASTER trial also showed improvements in sustained MRD negativity and progression free survival in patients with one HR FISH but not for those with two or more HR FISH [37]. ASCT seems to overcome the adverse prognosis of one HR FISH when paired with a potent induction regimen including an anti-CD38 antibody, second generation proteosome inhibitor and second generation immunomodulator. Dedicated trials for high-risk patients, in particular ultra-high-risk patients and plasma cell leukemia patients, are eagerly awaited [38, 39]. Therefore, the role of ASCT in HR MM warrants further study in prospective trials.

Thirdly, our results highlight the poor survival in those TE-unfit or refusing ASCT. Our study is unique in showcasing the poor survival outcomes of TE-refused patients that has not been reported before. Since upfront ASCT is a free service offered to all NDMM among TE patients in Hong Kong, ASCT refusal is most likely due to personal reasons or preference. Indeed, many socioeconomical factors closely shape and influence treatment choices of patients [18–21]. When patients refuse upfront ASCT, there should be an in-depth discussion of alternative treatment strategies and options. First, use of potent quadruplet induction regimens to increase the likelihood of achieving CR and MRD negativity in first remission should be explored [30, 31]. The results of the FORTE and the GMMG HD7 trials have shown that there may be a role of carfilzomib with the addition of an anti CD38 antibody for induction to enhance the depth of response even in non-transplant candidates [4, 40]. Second, the option of delayed ASCT can be discussed and if possible, early collection and storage of stem cells for future use [41]. Though upfront ASCT is associated with improved PFS compared to delayed ASCT, delayed ASCT does not compromise OS [42, 43] and should remain an option when upfront ASCT for personal or logistic reasons is not feasible [44]. Nonetheless, treating physicians should be aware that up to one-third of patients may be unable to receive ASCT at relapse due to development of new comorbidities, decline in performance status or rapid progression of disease at relapse [45].

Interestingly, our results showed that among ASCT recipients, OS was inferior in those ≥60 years than those <60. The cut-off at 60 years old was based on a previous retrospective analysis looking at whether age could affect outcomes of transplanted myeloma patients (≤66 years). In that study, there was a higher risk of death in those ≥60 years old, mainly due to the higher percentage of ISS 2/3 stages [46]. By contrast, IMWG-defined HR FISH was not over-represented in our patients ≥60 years of age [46–49].

For TE-unfit (renal) patients, the median creatinine was 368 umol/L, ranging from 125 to 1046 umol/L. Though there is increasing evidence ASCT can be safety performed in patients with severe renal impairment leading to similar benefits of PFS and OS as in patients with adequate renal function [50–53], the majority of our patients within this cohort received treatment before such practice was widely adopted with support of literature. The advancements in induction therapy with novel agents, use of melphalan 140 mg/m^2^ and improvements in peri-transplant supportive measures have undoubtedly played a major role in maximizing the effectiveness while minimizing the toxicities of ASCT for patients with renal impairment [54]. From a culture perspective, Chinese patients have a heavy stigma on renal dialysis and would avoid deterioration of renal function for fear of dialysis at all cost. Furthermore, it would be helpful to gather collective real-world data to evaluate whether the addition of an anti CD38 antibody on induction can salvage a higher percentage of renal impairment before ASCT is performed.

This study is limited by its retrospective design. As the decision for ASCT is based on the discretion of individual hematology centers, we do not have details of the exact medical comorbidities that precluded ASCT [55, 56]. As immunofixation was not performed routinely in all centers or SPE negative samples, distinction of CR and nCR was not possible in all cases, hence CR and nCR were grouped together for analysis. Indeed, deep responses of stringent CR conferred prognostic implications in both post induction and post ASCT settings [57].

Apart from reaffirming the role of ASCT, pretransplant risk factors and post-transplant responses, our study is the first to show the poor outcome of those refusing ASCT among the TE. The adverse impact of age ≥60-66 years among the transplanted was unaccounted for in our analysis. Finally, the impact of ASCT on HR MM warrants further randomized controlled studies.

## Data Availability

The datasets analyzed for this study are available from the corresponding author upon reasonable request.
